# Clinical Investigations of the Effect of* Citrus unshiu* Peel Pellet on Obesity and Lipid Profile

**DOI:** 10.1155/2018/4341961

**Published:** 2018-09-19

**Authors:** Seongil Kang, Sangyeol Song, Joosang Lee, Hyekyung Chang, Sanghun Lee

**Affiliations:** ^1^Jeju Institute of Korean Medicine, Republic of Korea; ^2^Department of Surgery, College of Medicine, Kyunghee University, Republic of Korea; ^3^Department of Medical Consilience, Graduate School, Dankook University, Republic of Korea

## Abstract

**Objectives:**

Several experimental studies have reported antiobesity and lipid-improving effects of* Citrus unshiu*. However, clinical studies on its effects are lacking. This study was designed to evaluate the impact of* Citrus unshiu *peel pellet (CUPP) on obesity and lipid profile.

**Methods:**

For 118 patients with body mass index (BMI) > 23 who took* Citrus unshiu* peel pellet (CUPP) for 4 weeks in a Public Health Center, laboratory and biometric readings before and after CUPP administration were analyzed.

**Results:**

Mean age of these subjects was 53.8±10.6 years (range: 18-75 years). There were 88 (74.6%) females in the study sample (n = 118). A significant (*p *< 0.01) decrease in BMI from 27.47±2.24 to 27.27±2.22 was observed in all subjects after CUPP treatment and 65.3% (N = 77) of them lost 1.03±0.83 kg of weight after 4 weeks of treatment. Total cholesterol level was significantly (*p *< 0.01) decreased from 204.0±37.4 mg/dL to 193.5±36.5 mg/dL. Significant (*p *< 0.05) decreases in levels of low-density lipoprotein, cholesterol, and triglyceride were also observed.

**Conclusions:**

These results suggest that CUPP in practice could help weight control and improve total cholesterol level. Findings of this study provide clinical foundation for future large-scale trials to establish clinical benefits of CUPP.

## 1. Introduction

Obesity and related hyperlipidemia have become major worldwide health problems. With sharply increasing incidence of metabolic disease and associated morbidity and mortality, both medical and economical costs are also expected to increase [1]. Nowadays, synthetic drugs to lower weight or serum lipid in obese patients with hyperlipidemia are available. However, they have limitations such as adverse effects and progression of drug resistance [2]. In this regard, application of medicinal plants is necessary.

Citrus flavonoids as polyphenolic compounds have emerged as promising therapeutic agents for the treatment of obesity, insulin resistance, and dyslipidemia [3, 4]. Flavonoids such as naringin, hesperidin, and nobiletin have been experimentally proven to be able to lower lipid levels with insulin-sensitizing and anti-inflammatory properties primarily through inhibition of hepatic fatty acid synthesis and induction of increased fatty acid oxidation [5–8].* Citrus aurantium* has been used as one of common dietary supplements for weight loss in the US because it increases energy expenditure and lipolysis and acts as a mild appetite suppressant [9, 10]. Synephrine, its proposed active constituent, can mimic the action of epinephrine and norepinephrine as alpha-adrenergic agonists. However, human clinical studies have demonstrated that naringin or hesperidin supplement exerts no effect on lipid profile [11, 12]. Active compound isolated from herbs rarely demonstrates high pharmacological activity against metabolic dysregulation because there is no synergistic interaction or multifactorial effects between various compounds present in herbal extract [13]. Therefore, clinical study of citrus extract itself, not isolated flavonoid on the metabolic diseases, is required.

Dried peel of* Citrus unshiu *Markov has been traditionally used as a popular herbal ingredient for treatment of various digestive dysfunctions, including abdominal distension, nausea, vomiting, and dyspepsia in East Asia such as Korea and Japan [14]. Based on its excellent properties and safe use, this herb has been commonly used for a long time in food or medicine preparation which was registered and managed as policy documents of Korea Food and Drug Administration [15, 16]. The dried peel of* Citrus unshiu*, like other citrus species, has been experimentally proven to be able to ameliorate hyperglycemia through inhibition of hepatic gluconeogenic enzyme and induction of insulin/glucagon secretion. It can also ameliorate hypertriglyceridemia via inhibition of lipid absorption and lipogenesis. In addition, it promotes lipolytic effects in the liver [17–20]. However, clinical studies on the effect of* Citrus unshiu* on obesity and lipid metabolism are insufficient [21]. Therefore, the objective of this study was to explore the efficacy and safety of* Citrus unshiu* in overweight or obese subjects. Results of this study will provide a clinical foundation for future large-scale clinical trials.

## 2. Patients and Methods

### 2.1. Subjects and Ethics

The Public Health Center in Jejusi, Republic of Korea, has provided a variety of weight management programs including exercise, diet, and behavior modification to local residents. Herbal medicines were also provided to those who did not lose weight after such effort with voluntary consent. According to the Asia-Pacific obesity classification, all subjects were classified into five different groups: underweight (< 18.5 kg/m^2^), normal weight (18.5 to 22.99 kg/m^2^), overweight (23 to 24.99 kg/m^2^), class I obese (25 to 29.99 kg/m^2^), and class II obese (≥ 30 kg/m^2^) [22]. Therefore, all 157 adult patients with BMI greater than 23 kg/m^2^ were treated with* Citrus unshiu* peel pellet (CUPP) for 4 weeks between September and October 2017. After excluding cases with missing data on anthropometric and biochemical parameters before and after CUPP administration, 118 patients were finally analyzed. This project was approved by the Institutional Review Board of Dankook University (DKU 2017-11-001). It was conducted in accordance with the Declaration of Helsinki.

### 2.2. *Citrus unshiu* Peel Pellet (CUPP) and Treatment Course

CUPP was prepared from dried peels of* Citrus unshiu* cultivated organically in Jeju Island, Republic of Korea. After juice extraction process,* Citrus unshiu *peels were collected and dried in an air-oven at 50°C. Subsequently, they were crushed and sieved to particle size of 0.25 mm. They then underwent agglomeration to form larger pellet size of 5 mm.

**Figure 1:**
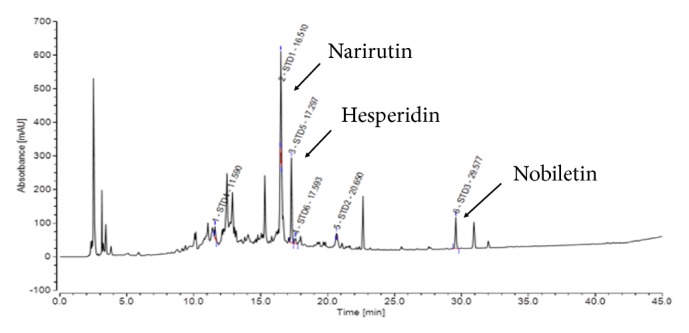
Major constituents of* Citrus unshiu *peel pellet (CUPP) revealed by HPLC.

For analysis of CUPP components, 10 grams of the final CUPP was extracted with 10 ml of 80% methanol under ultrasonication for 30 min and centrifuged at 3000 rpm for 5 min. The supernatant was filtered through a Whatman No. 1 filter paper. The filtrate was concentrated using a vacuum rotary evaporator (Büchi R-100, Germany) and lyophilized using a freeze dryer (Ilshin Biobase, Korea). The extract was dissolved in high-performance liquid chromatography (HPLC) grade methanol to a final concentration of 10 mg/mL. The solution was filtered through a 0.45-*μ*m membrane and 20 *μ*L of the filtrate was subjected to HPLC analysis. HPLC profile of CUPP is shown in Figure 1, showing the presence of narirutin, hesperidin, and nobiletin. The quality of CUPP was tested and controlled according to quality standards of the Korea Food and Drug Administration (hesperidin content > 4.0%). The proximate composition of CUPP following AOAC method was carbohydrate (82.4%), sugar (18.9%), crude protein (5.8%), crude fat (0.0%), and caloric value (353.2 kcal/100 g). Daily oral administration of 18 mg (6 mg three times a day) of CUPP was prescribed.

### 2.3. Protocols and Procedures

Demographic and clinical variables including gender, age, waist circumference, weight, height, and medical comorbidities were collected from patients. Waist circumference was measured at the midway between the last rib and the iliac crest. BMI was calculated as kg/m^2^. Body composition, particularly the amount of body fat, was measured by multifrequency tetrapolar bioelectrical impedance method (InBody230®, Biospace, Seoul, South Korea) after at least 8 hours of fasting. Fasting blood samples were also obtained for laboratory analysis of aspartate aminotransferase (AST), alanine aminotransferase (ALT), *γ*-glutamyltranspeptidase (*γ*-GTP), total cholesterol (TC), triglyceride (TG), high-density lipoprotein (HDL) cholesterol, and low-density lipoprotein (LDL) cholesterol levels. These clinical data including anthropometric and biochemical parameters were repeatedly measured after 4 weeks of CUPP treatment under similar fasting conditions. Medications for commodities were not changed during the 4 weeks of CUPP treatment. Recorded adverse effects during CUPP administration were also checked.

### 2.4. Statistical Analyses

Baseline demographic and clinical characteristics of patients are reported as mean ± standard deviation for continuous variables and as frequencies and percentages for categorical variables. Differences in anthropometric and biochemical parameters between groups were examined by Student's t-test. Paired samples t-test was used to evaluate the significance and difference in data obtained from quantitative laboratory and biometric tests before and after treatment with CUPP. Pearson correlation analysis was performed for weight loss and changes in TC level. Statistical significance was considered when *p* value was equal to or less than 0.05. All statistical analyses were carried out using R, a free package from the R Foundation for Statistical Computing.

## 3. Results

The study population was composed of 30 males and 88 females with a mean age of 53.8±10.6 years (range: 18-75 years) and a median BMI of 27.1 kg/m^2^ (range: 23.4-36.1 kg/m^2^). Around a third of these subjects (N = 38, 32.2%) were taking antihypertension medication along with dyslipidemia medication including statin (N = 29, 24.6%) or oral hypoglycemic drugs for type 2 diabetes (N = 7, 5.9%). With respect to smoking history, there were five (4.2%) current smokers, 13 (11.0%) former smokers, and 100 (84.7%) never-smokers. Regarding alcohol consumption, there were 66 (55.9%) current drinkers, 5 (4.2%) former drinkers, and 47 (39.8%) never-drinkers.

Main characteristics of anthropometric and biochemical measurements between baseline and 4th week after CUPP administration are summarized in Table 1. BMI, weight, and waist circumference were significantly decreased after CUPP treatment. However, the reduction volume of BMI was only about 0.2 kg/m^2^ with weight reduction of 500 grams during 4 weeks. There was no change in fat mass based on bioelectrical impedance method. However, 77 (65.3%) responders experienced weight loss after CUPP treatment, amounting to 1.03±0.83 kg in 4 weeks.

**Figure 2:**
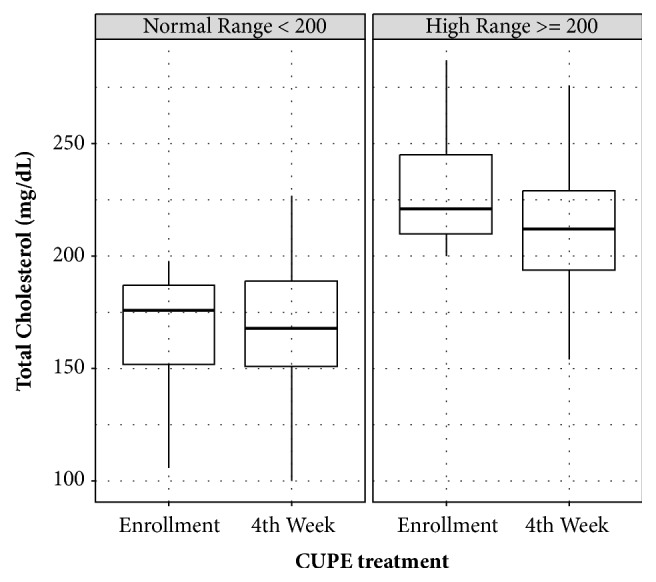
Total cholesterol changes in subjects with normal range (< 200 mg/dL) and abnormal range (≥ 200 mg/dL) of cholesterol before CUPP treatment, showing definite improvement in the abnormal group after treatment.

**Figure 3:**
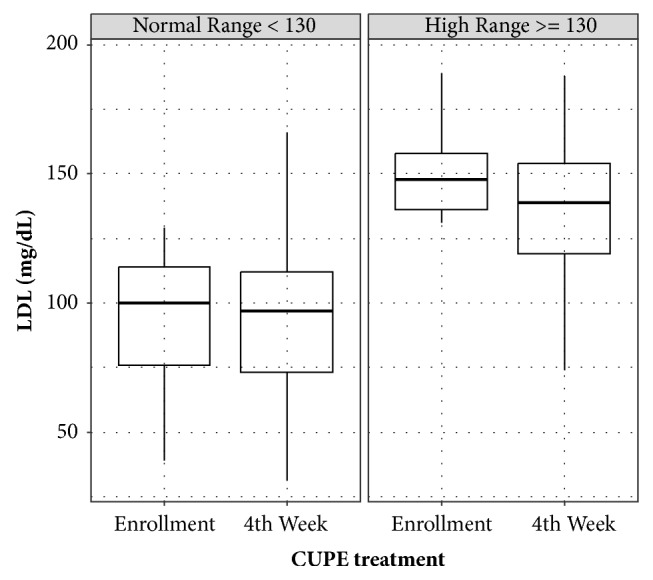
Changes in low-density lipoprotein (LDL) in subjects with normal range (< 130 mg/dL) and abnormal range (≥ 130 mg/dL) of LDL before CUPP treatment, showing definite improvement in the abnormal group after treatment.

**Figure 4:**
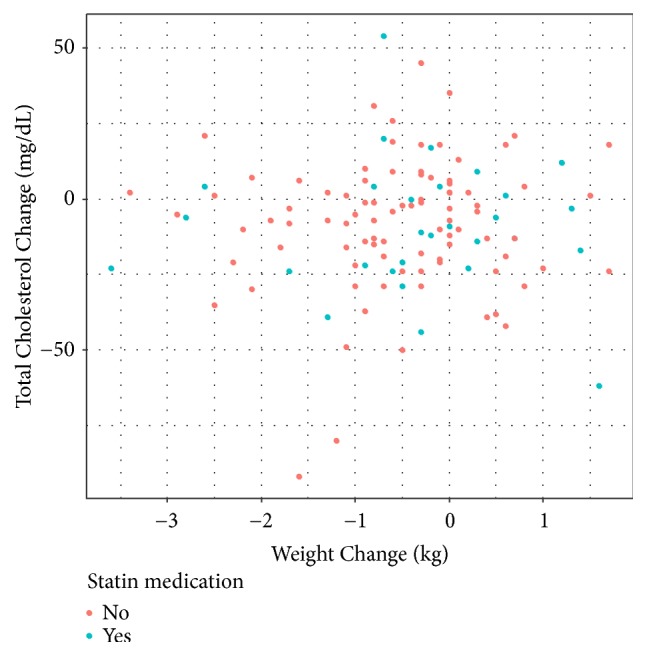
Total cholesterol versus weight change during CUPP treatment based on taking or not taking statin medication (correlation coefficient r = 0.0597, *p* = 0.521).

Based on laboratory tests, TC exhibited significantly more decrease compared to other lipid levels. This could be explained by LDL cholesterol reduction, but not HDL cholesterol reduction. Subanalysis after separating normal and abnormal TC or LDL cholesterol range also demonstrated that TC and LDL cholesterol levels in the abnormal group were decreased more by CUPP treatment compared to those in the normal group (Figures 2 and 3). Moreover, TC reduction subsequent to CUPP treatment was independent of statin medications (*p* = 0.797) or weight loss (r = 0.0597, 95% CI: −0.122 to 0.238, *p* = 0.521, Figure 4), suggesting that CUPP treatment could complement statin with different mechanism of action. AST and ALT levels were also significantly decreased below their normal ranges. This suggests that CUPP treatment is safe for the liver.

Adverse events related to CUPP treatment were not serious. Gastrointestinal discomfort (N = 19, 16.1%), mild diarrhea (N = 8, 6.8%), headache (N = 1, 0.8%), and dizziness (N = 1, 0.8%) were reported which resolved later without any intervention.

## 4. Discussion

Compounds present in CUPP are narirutin, its isomer naringin, hesperidin, and nobiletin. They have been proven to exert antiobesity effects both* in vitro* and* in vivo *[5–8]. Furthermore, these compounds have been found to display strong anti-inflammatory and antioxidant activities [23]. A number of molecular mechanisms particularly underlying lipid metabolism in obesity have been explained [3, 4]. It has been reported that hesperidin possesses inhibitory activity against lipase, resulting in inhibition of fat absorption [6]. Nobiletin can improve obesity and insulin resistance in rats fed a high-fat diet by increasing PPAR*γ* expression or regulating NF-*κ*B and Nrf2 pathways [7, 24]. However, therapeutic uses of these flavonoids are significantly limited due to the lack of adequate clinical evidence.

Peel extracts of* Citrus unshiu* have shown antiobesity effects through inhibition of lipogenesis and adipogenesis in both normal and high-fat diet fed animal models compared to peel extracts of other citrus such as* Citrus aurantium *L. which does not demonstrate any improvement in obesity in normal diet fed mice [17, 20, 25, 26]. The preventive effect of* Citrus unshiu* on atrophy of skeletal muscle and weight loss by suppressing systemic inflammation and production of procachectic factors in tumor-bearing mice has been reported [27]. Our results showed that CUPP has antiobesity effect after 4 weeks of treatment, with 500 grams of reduction in weight of obese subjects. Previous studies on* Citrus unshiu* have shown its antiobesity effect after 9 weeks of administration in high-fat diet fed Sprague-Dawley male rats as well as after 5 or 6 weeks of administration in normal diet fed mouse [17, 20]. Therefore, it has been proposed that the observation period should be longer than 4 weeks to notice sufficient effect of CUPP on weight loss.

Effects of* Citrus unshiu* on lowering cholesterol and TG have been repeatedly confirmed in many preclinical studies [17, 19, 28]. A mixture of naringin and hesperidin can also significantly lower levels of plasma and hepatic cholesterol and TG levels as well as 3-hydroxy-3-methylglutaryl-CoA (HMG-CoA) reductase activity in rats [29]. However, in human studies, citrus flavonoids such as naringin (500 mg/day) or hesperidin (800 mg/day) did not exhibit any lipid-lowering activity on TC, LDL cholesterol, or TG levels after 4 weeks of administration in moderately hypercholesterolemic individuals [11]. Another clinical study has demonstrated that peel extract of* Citrus unshiu* could significantly suppress elevated TG level, but not TC level [21]. Our study showed that CUPP administration for only 4 weeks significantly decreased TC, LDL cholesterol, and TG concentrations in plasma. The lipid-lowering mechanism of* Citrus unshiu *is suggested as follows. Reduced hepatic steatosis along with increased hepatic insulin sensitivity and fatty acid oxidation can promote apolipoprotein B degradation, thereby contributing to lowering the secretion of LDL and LDL particle and circulating cholesterol level [3]. The beneficial effect of* Citrus unshiu *is associated with 5′-adenosine monophosphate-activated protein kinase activation in adipose tissue [30]. Additionally,* Citrus unshiu *could change microbiota in the colon. The white part of the peel from* Citrus unshiu* can decrease serum TG due to increased levels of bifidobacteria in rat cecum [31].

Our study population was chiefly middle-aged women experiencing estrogen hormone changes at menopause, a condition well known to be associated with obesity and hyperlipidemia [32]. The peel of* Citrus unshiu *can significantly lower TC and TG concentrations in the liver of ovariectomized rats showing obvious lipid metabolic disturbance [28]. Therefore, it was hypothesized that CUPP could be more beneficial for individuals with dyslipidemia and those who were suffering from a reduction in estrogen levels. In our study, lipid-lowering effect of CUPP treatment was independent of weight loss or statin medications. Therefore, CUPP could help the condition of dyslipidemia in individuals with lean body mass or in patients whose lipid levels remain uncontrolled even by statin medication. CUPP treatment is tolerable with only minor nonhematologic adverse effects including gastrointestinal discomfort (16.1%) and mild diarrhea (6.8%). In some cases, improvement in constipation was observed. It could be explained by increased gastrointestinal motility by CUPP based on a preclinical study [14]. None of our patients discontinued CUPP treatment due to CUPP-related adverse events.

This study has several limitations that should be considered. Firstly, this study was planned as part of a health promotion program at a Public Health Center, not for clinical trial. Thus, the absence of a control group to make a comparison led to careful interpretation of results. However, most of enrolled subjects had failed in weight loss in spite of a variety of weight management programs including exercise, diet, and behavior modification previously provided by the Public Health Center for more than 6 months. Therefore, the improvement of weight circumference, weight, or total cholesterol without lifestyle changes could be interpreted by the effect of CUPP treatment. Secondly, the observation period was only for 4 weeks, which is relatively short compared to most intervention trials on obesity. If CUPP treatment would be administered for a longer observation period, the effect of weight loss could be even greater. Finally, there was no inquiry of daily intake showing the changes of calorie intake. Therefore, it is difficult to distinguish whether the effect of CUPP treatment is due to a decrease in the amount of diet through loss of appetite or an increase of the basal metabolic rate which were suggested by the mechanism of action in Citrus flavonoids.

In conclusion, this study shows that CUPP treatment is well tolerated in overweight or obese patients for weight control. It can improve lipid levels in those with moderate dyslipidemia either alone or in combination with statin. Therefore, our results encourage further studies on the lipid-lowering effect of CUPP or its flavonoids due to low toxicity and different mechanism of action compared to conventional statin medication. Additional randomized and well-controlled multicenter clinical trials involving large populations are necessary to evaluate the efficacy and safety of CUPP in the treatment of obesity and dyslipidemia.

## Figures and Tables

**Table 1:** Changes in clinical variables after 4 weeks of CUPP treatment (N = 118).

	Baseline	4th Week	*p*
Waist circumference (cm)	91.53±7.55	90.36±7.27	0.0002
Weight (kg)	71.09±9.52	70.59 ±9.40	< 0.0001
BMI (kg/m^2^)	27.47±2.24	27.27±2.22	< 0.0001
Body fat mass (kg)	24.98±4.78	24.87±4.61	0.481
Fat free mass (kg)	46.11±8.57	45.68±8.84	0.023
Systolic blood pressure (mmHg)	128.9±16.6	126.9 ±14.8	0.554
Diastolic blood pressure (mmHg)	81.4±10.8	81.9±10.7	0.558
Total cholesterol (mg/dL)	201.5±37.4	192.8±36.5	< 0.0001
LDL cholesterol (mg/dL)	117.3±34.5	111.8±34.6	0.011
HDL cholesterol (mg/dL)	56.3±14.6	55.8±13.2	0.482
Triglyceride (mg/dL)	139.7±78.0	126.1±74.5	0.026
AST (U/L)	24.1±8.1	22.4±8.1	0.0093
ALT (U/L)	23.3±12.3	21.2±11.6	0.0167
*γ*-GTP (U/L)	29.9 ±38.5	32.0 ±35.9	0.153

BMI: body mass index; AST: aspartate aminotransferase; ALT: alanine aminotransferase; and *γ*-GTP: *γ*-glutamyltranspeptidase.

## Data Availability

The data used to support the findings of this study are available at http://jikom.or.kr/.

## References

[1] Finkelstein E. A., Trogdon J. G., Cohen J. W., Dietz W. (2009). Annual medical spending attributable to obesity: payer- and service-specific estimates. *Health Affairs*.

[2] Bang C. N., Okin P. M. (2014). Statin Treatment, New-Onset Diabetes, and Other Adverse Effects: A Systematic Review. *Current Cardiology Reports*.

[3] Assini J. M., Mulvihill E. E., Huff M. W. (2013). Citrus flavonoids and lipid metabolism. *Current Opinion in Lipidology*.

[4] Nakajima V. M., Macedo G. A., Macedo J. A. (2014). Citrus bioactive phenolics: Role in the obesity treatment. *LWT- Food Science and Technology*.

[5] Alam M. A., Subhan N., Rahman M. M., Uddin S. J., Reza H. M., Sarker S. D. (2014). Effect of citrus flavonoids, naringin and naringenin, on metabolic syndrome and their mechanisms of action. *Advances in Nutrition*.

[6] Kawaguchi K., Mizuno T., Aida K., Uchino K. (1997). Hesperidin as an inhibitor of lipases from porcine pancreas and pseudomonas. *Bioscience, Biotechnology, and Biochemistry*.

[7] Yoshigai E., Machida T., Okuyama T. (2013). Citrus nobiletin suppresses inducible nitric oxide synthase gene expression in interleukin-1*β*-treated hepatocytes. *Biochemical and Biophysical Research Communications*.

[8] Jia J., Yao W., Guan M. (2011). Glucocorticoid dose determines osteocyte cell fate. *The FASEB Journal*.

[9] Haaz S., Fontaine K. R., Cutter G., Limdi N., Perumean-Chaney S., Allison D. B. (2006). Citrus aurantium and synephrine alkaloids in the treatment of overweight and obesity: an update. *Obesity Reviews*.

[10] Stohs S. J., Preuss H. G., Shara M. (2012). A review of the human clinical studies involving citrus aurantium (bitter orange) extract and its primary protoalkaloid p-synephrine. *International Journal of Medical Sciences*.

[11] Demonty I., Lin Y., Zebregs Y. E. M. P. (2010). The citrus flavonoids hesperidin and naringin do not affect serum cholesterol in moderately hypercholesterolemic men and women. *Journal of Nutrition*.

[12] Jung U. J., Kim H. J., Lee J. S. (2003). Naringin supplementation lowers plasma lipids and enhances erythrocyte antioxidant enzyme activities in hypercholesterolemic subjects. *Clinical Nutrition*.

[13] Wagner H., Ulrich-Merzenich G. (2009). Synergy research: approaching a new generation of phytopharmaceuticals. *Phytomedicine*.

[14] Lyu J. H., Lee H.-T. (2013). Effects of dried Citrus unshiu peels on gastrointestinal motility in rodents. *Archives of Pharmacal Research*.

[15] Seo J., Lim H., Chang Y.-H. (2015). Effects of jeju citrus unshiu peel extracts before and after bioconversion with cytolase on anti-inflammatory activity in RAW264.7 cells. *Journal of the Korean Society of Food Science and Nutrition*.

[16] Lee S., Suh S., Lee K., Yang J., Choi S., Park S. (2013). Anti-Inflammatory Effect of Peel Extracts from Citrus Fruits. *Journal of Food Hygiene and Safety*.

[17] Jung S. (2013). *Anti-obesity effect of Citrus unshiu peel in rats fed a high-fat diet*.

[18] Kim G. N., Shin M. R., Shin S. H. (2016). Study of Antiobesity Effect through Inhibition of Pancreatic Lipase Activity of Diospyros kaki Fruit and Citrus unshiu Peel. *BioMed Research International*.

[19] Lim H., Yeo E., Song E. (2015). Bioconversion of Citrus unshiu peel extracts with cytolase suppresses adipogenic activity in 3T3-L1 cells. *Nutrition Research and Practice*.

[20] Park H.-J., Jung U. J., Cho S.-J., Jung H.-K., Shim S., Choi M.-S. (2013). Citrus unshiu peel extract ameliorates hyperglycemia and hepatic steatosis by altering inflammation and hepatic glucose- and lipid-regulating enzymes in db/db mice. *The Journal of Nutritional Biochemistry*.

[21] Fritzsch H. (2011). Composite weak bosons, leptons and quarks. *Modern Physics Letters A*.

[22] Weisell R. C. (2002). Body mass index as an indicator of obesity. *Asia Pacific Journal of Clinical Nutrition*.

[23] Chen X.-M., Tait A. R., Kitts D. D. (2017). Flavonoid composition of orange peel and its association with antioxidant and anti-inflammatory activities. *Food Chemistry*.

[24] Lee Y.-S., Cha B.-Y., Choi S.-S. (2013). Nobiletin improves obesity and insulin resistance in high-fat diet-induced obese mice. *The Journal of Nutritional Biochemistry*.

[25] Arbo M. D., Schmitt G. C., Limberger M. F. (2009). Subchronic toxicity of Citrus aurantium L. (Rutaceae) extract and p-synephrine in mice. *Regulatory Toxicology and Pharmacology*.

[26] Yang G., Lee J., Jung E.-D., Ham I., Choi H.-Y. (2008). Lipid lowering activity of Citri unshii pericarpium in hyperlipemic rats. *Immunopharmacology and Immunotoxicology*.

[27] Kim A., Im M., Gu M. J., Ma J. Y. (2016). Citrus unshiu peel extract alleviates cancer-induced weight loss in mice bearing CT-26 adenocarcinoma. *Scientific Reports*.

[28] Lim D. W., Lee Y., Kim Y. T. (2014). Preventive effects of citrus unshiu peel extracts on bone and lipid metabolism in OVX rats. *Molecules*.

[29] Bok S.-H., Lee S.-H., Park Y.-B. (1999). Plasma and hepatic cholesterol and hepatic activities of 3-hydroxy-3- methyl-glutaryl-CoA reductase and acyl CoA: cholesterol transferase are lower in rats fed citrus peel extract or a mixture of citrus bioflavonoids. *Journal of Nutrition*.

[30] Karagozlu M. Z., Kim M., Lee M. (2016). Citrus Peel Ethanol Extract Inhibits the Adipogenesis Caused from High Fat-Induced DIO Model. *Journal of Food and Nutrition Sciences*.

[31] Iwata E., Hotta H., Goto M. (2012). Hypolipidemic and bifidogenic potentials in the dietary fiber prepared from Mikan (Japanese mandarin orange: Citrus unshiu) albedo. *Journal of Nutritional Science and Vitaminology*.

[32] Polotsky H. N., Polotsky A. J. (2010). Metabolic implications of menopause. *Seminars in Reproductive Medicine*.

